# Effects of virtual reality with different modalities on upper limb recovery: a systematic review and network meta-analysis on optimizing stroke rehabilitation

**DOI:** 10.3389/fneur.2025.1544135

**Published:** 2025-04-01

**Authors:** Jiali Zhang, Mingxiu Liu, Junlin Yue, Jinmei Yang, Yan Xiao, Jie Yang, Enli Cai

**Affiliations:** ^1^The Third Affiliated Hospital of Yunnan University of Chinese Medicine, Kunming Municipal Hospital of Traditional Chinese Medicine, Kunming, Yunnan, China; ^2^Bishan Hospital of Chongqing Medical University, Chongqing, China; ^3^College of Nursing, Yunnan University of Chinese Medicine, Kunming, Yunnan, China

**Keywords:** stroke, upper limb, rehabilitation, virtual reality, network meta-analysis, systematic review

## Abstract

**Background:**

As a major cause of disability worldwide, stroke affects about 80% of survivors with upper limb (UL) motor dysfunction, significantly impairing their quality of life. Virtual reality (VR) has been recognized as an innovative rehabilitation tool; however, the effectiveness of VR systems with different immersion modalities is still uncertain. This systematic review and network meta-analysis (NMA) aims to evaluate the comparative effectiveness of intervention measures, including non-immersive gaming consoles, immersive VR (IVR), non-immersive VR (NIVR), and conventional therapy (CT) on upper limb motor function in stroke rehabilitation.

**Materials and methods:**

A systematic search of PubMed, Embase, Cochrane Library, and Scopus identified randomized controlled trials (RCTs) published up to 12 June 2024. UL motor recovery was assessed using the Fugl-Meyer Upper Extremity (FMUE) scale. The NMA was performed using the Bayesian approach with the BUGSnet package in R software to calculate the relative effectiveness of each intervention.

**Results:**

34 RCTs involving 1,704 participants were included. Among non-immersive gaming systems, Microsoft Kinect demonstrated the greatest effective in enhancing UL motor function, followed by Nintendo Wii, then NIVR and IVR head-mounted devices. CT showed the least effective. Specifically, Microsoft Kinect significantly improved FMUE scores (mean difference [MD] = 7.27, 95% confidence interval [CI]: 0.59 to 13.77, *p* < 0.05), followed by Nintendo Wii (MD = 4.53, 95% CI: 0.87 to 8.14, *p* < 0.05), and NIVR (MD = 3.57, 95% CI: 1.18 to 6.01, *p* < 0.05). In contrast, IVR head-mounted devices showed no statistically significant differences in outcomes, with MD of 4.16 (95% CI: −0.02 to 8.38).

**Conclusion:**

Non-immersive gaming console of Microsoft Kinect is the most effective intervention for improving UL motor function in stroke survivors. In contrast, IVR head-mounted devices did not offer significant advantages over CT. These findings suggest that non-immersive gaming consoles of Microsoft Kinect could be a more cost-effective and accessible alternative for stroke rehabilitation.

## 1 Introduction

Stroke represents a leading cause of global disability, impacting nearly 14 million individuals each year (1, 2). Although stroke-related mortality rates exhibit a downward trajectory, the prevalence of stroke survivors with long-term sequelae is increasing. This rise is primarily due to population growth and aging (3). As a result, the prevalence of persistent disabilities among adult populations is growing (4), which is increasing the demand for rehabilitation. Data from the Global Burden of Disease study indicate that over 2.4 billion people worldwide required rehabilitation services in 2019, with a significant proportion being stroke survivors with motor impairments (5). Among the various sequelae, approximately 80% of stroke survivors experience UL motor impairments. These impairments vary and often manifest as reduced hand grip strength, diminished finger dexterity, and limited elbow and shoulder mobility (6). Such deficits severely impact daily functioning, making it difficult for patients to perform tasks such as dressing, eating and maintaining personal hygiene. As a result, stroke survivors often experience a decline in autonomy and quality of life (7). Moreover, these impairments can increase dependence on others and lead to psychological distress and social isolation (8). Rehabilitation of UL motor function in stroke patients requires ongoing, dedicated efforts over an extended period. However, traditional rehabilitation approaches often encounter barriers like resource limitations and insufficient patient engagement (9), which lead to rehabilitation outcomes, emphasizing the need for innovative interventions. VR has emerged as a promising therapeutic modality to address these challenges. By providing a highly realistic three-dimensional environment, VR enables patients to engage in simulated activities. Through the integration of visual, auditory, and tactile elements, VR provides real-time feedback to patients (10, 11). Additionally, VR may also facilitate functional recovery by activating mechanisms of neuroplasticity (12).

In the field of post-stroke UL functional recovery, VR technologies encompass a variety of applications. These primarily include IVR, such as head-mounted devices, NIVR, and non-immersive gaming consoles (e.g., Nintendo Wii and Microsoft Kinect) (13, 14). While IVR provides high immersion levels, which may enhance motor recovery (15, 16), its widespread adoption is limited by challenges such as high costs and patient discomfort (17). NIVR, in contrast, is more accessible but has shown variability in its effectiveness due to differences in technological features and interactivity (18, 19).

Many existing studies focus on individual VR modalities, leaving a gap in direct comparisons of different VR technologies for UL rehabilitation (20, 21). A 2022 NMA (22) suggested the potential benefits of IVR for UL motor recovery. However, it did not incorporate recent advances in VR technologies or newly published RCTs. Moreover, the study did not consider the practical limitations of IVR, which have prompted a growing interest in NIVR systems for scalable stroke rehabilitation. Based on these considerations, we hypothesize that non-immersive gaming consoles, such as Microsoft Kinect, may offer a superior effect due to their accessibility, affordability, and ease of integration into clinical and home-based rehabilitation. Therefore, we will test this through a systematic review and NMA to provide a comprehensive comparison of VR interventions with varying levels of immersion. By doing so, it offers updated evidence to guide clinical decision-making and enhance rehabilitation strategies for stroke survivors.

## 2 Materials and methods

### 2.1 Design

This systematic review and NMA was conducted in accordance with the guidelines provided by the Preferred Reporting Items for Systematic Reviews and Meta-Analyses statement (PRISMA-NMA) (23). The study was registered with PROSPERO (CRD42024610482), which can be found at https://www.crd.york.ac.uk/PROSPERO/view/CRD42024610482.

### 2.2 Search methods

A systematic search was performed across PubMed, Embase, the Cochrane Library, and Scopus from their inception up to 12 June 2024. The search strategies were tailored for each database, combining controlled vocabulary terms and title/abstract keywords related to virtual reality, upper limb, stroke, and randomized controlled trials. The comprehensive search strategies are detailed in Supplementary Table S1. The literature review was restricted to peer-reviewed journal articles reported human studies in English.

### 2.3 Eligibility criteria

Studies were eligible for our NMA if they met all following criteria:

1. Study design: RCTs.
2. Patient characteristics: adult individuals experiencing their initial ischemic or hemorrhagic stroke, with diagnosis established according to well-defined or internationally recognized diagnostic criteria, and with no restrictions on race or gender.
3. Intervention: VR-based rehabilitation, encompassing IVR (such as head-mounted devices), NIVR, and non-immersive gaming consoles (such as Microsoft Kinect or Nintendo Wii).
4. Comparison: CT, including conventional rehabilitation, occupational therapy, physical therapy, usual care alone, and UL conventional. The frequency and duration should correspond to those of the intervention group.
5. Outcome: FMUE was used to assess upper extremity function.

Studies were excluded based on the following criteria:

1. VR was integrated with robot-assisted therapy or neuromodulation in the intervention.
2. The total treatment dosages administered to the VR group and the CT group were not equivalent.
3. Studies with an intervention frequency lower than twice weekly and lacking clear specification of individual intervention durations.
4. Studies with unclear outcome indicators that could not be accurately extracted, or those with missing data.
5. Articles with only abstracts, reviews, case reports and study protocols.
6. In instances of duplicate published studies, the research presenting the most comprehensive data was chosen for inclusion.

### 2.4 Data extraction

Two independent reviewers evaluated studies for inclusion through a two-step process: initially screening titles and abstracts, followed by a comprehensive full-text assessment based on predetermined inclusion and exclusion criteria to determine final eligibility. Any disagreements were resolved through consultation with a third reviewer or by referencing to the original study. Data extraction was conducted using a standardized form to collect essential information, including the first author, publication year, study location, intervention and comparison group content and dosage, sample size, age, male proportion, and assessment time points for evaluating VR effects. Furthermore, elements critical for risk of bias assessment were recorded. In studies featuring multiple groups, only the CT group with a matched treatment dosage matching the VR group was included for comparison. For studies presenting both Per-protocol and Intention-to-Treat (ITT) analyses, ITT data were prioritized for extraction (24).

### 2.5 Assessment of the risk of bias

Version 2 of the Cochrane risk-of-bias tool for randomized trials (RoB-2) was employed to evaluate the quality of included studies (25). Two independent reviewers assessed six domains: bias in the randomization process, bias due to deviations from intended interventions, bias from missing outcome data, bias in outcome measurement, bias in selection of reported results, and overall bias. The studies were categorized as low risk of bias (all domains low risk), some concerns (concerns in at least one domain but no high risk), or high risk of bias (high risk in at least one domain). Discrepancies between reviewers were resolved through discussion to reach a consensus.

### 2.6 Statistical analysis

This study employed a Bayesian NMA to evaluate continuous outcome measures associated with UL function recovery in stroke patients. All statistical analyses were conducted using R (version 4.4.1; R Foundation for Statistical Computing, Vienna, Austria) with the “BUGSnet” package (26). We employed Bayesian NMA to model the posterior probability distribution, providing insights into the relative effects of treatments, quantifying uncertainty around parameter estimates and the ranking of the treatments within the network. The analysis employed a Markov Chain Monte Carlo (MCMC) with Gibbs sampling for robust parameter estimation (27), utilizing a Bayesian framework with 1,000 adaptations, 10,000 burn-ins, and 50,000 iterations. Continuous outcome data were presented as mean differences (MD) with corresponding 95% credible intervals; *p* < 0.05 suggests a statistically significant difference in outcome measures (28). Transitivity in this NMA was addressed by including studies with comparable patient populations, intervention designs, and outcome measures. Model selection and goodness-of-fit were evaluated through deviance information criteria (DIC), with lower values indicating superior model fit (29). Model adequacy was further evaluated by comparing the residual deviance with the number of unconstrained data points, where a close alignment suggested an adequate fit (30). Convergence was assessed using the Gelman–Rubin–Brooks diagnostic, with a potential scale reduction factor (PSRF) value below 1.05 considered indicative of acceptable convergence (31).

Furthermore, surface under the cumulative ranking (SUCRA) values were employed to rank the various interventions, with higher SUCRA values signifying more effective interventions (32). SUCRA values approach one when a treatment consistently ranks first, and approach zero when it consistently ranks last (33). Following this analysis, we developed the league table and heat plot, providing comprehensive information on the relative effectiveness and associated uncertainty for all possible intervention pairs.

## 3 Results

### 3.1 Study selection

**Figure 1:**
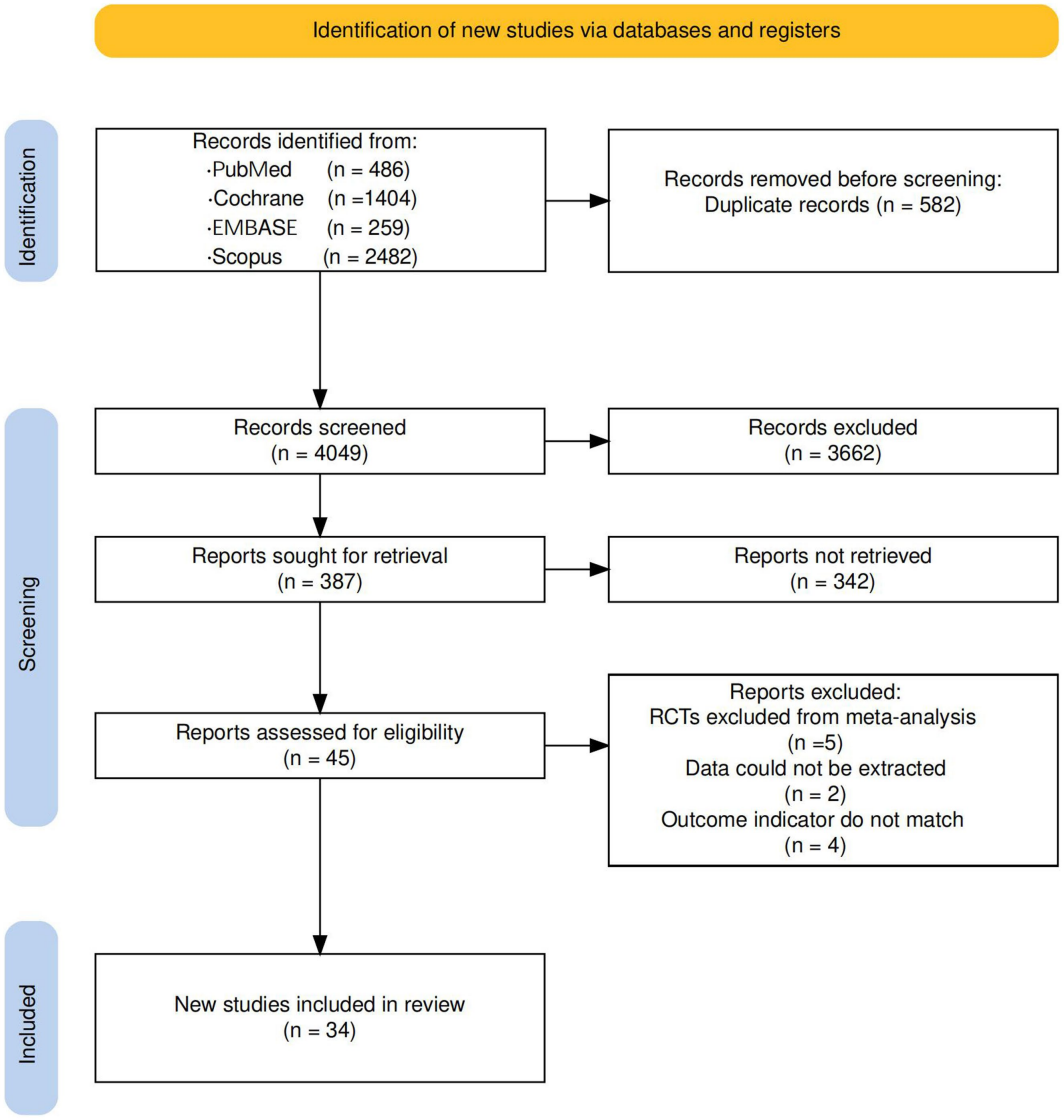
PRISMA flow diagram illustrating the process for searching and selecting eligible studies included in the network meta-analysis.

The search strategy initially identified 4,631 studies from the databases. After eliminating duplicates, 4,049 studies remained for title and abstract screening. Subsequently, 387 articles underwent full-text assessment based on predefined inclusion and exclusion criteria. Ultimately, 34 RCTs met the eligibility criteria and were included in this systematic review and NMA (34–67). The PRISMA flow diagram is depicted in Figure 1.

### 3.2 Characteristics of included studies

The current analysis encompasses 34 RCTs were included, all of which were two-arm studies, involving a combined total of 1,704 participants. Table 1 provides a summary of the characteristics of these studies. Across included studies, the frequency, duration, and total dose of interventions were comparable between VR and CT groups. The intervention duration ranged from 2 to 16 weeks, with the total VR intervention dose spanning 7 to 56 h. All studies utilized the Fugl-Meyer assessment to evaluate UL function. Each study employed two measurement time points: baseline and post-intervention. Follow-up evaluations were conducted in some studies to assess long-term effects. However, the follow-up time varied, with the longest being 12 weeks post-intervention (45, 55) and the shortest being 3 weeks post-intervention (41). One study (40) conducted two follow-ups: at 4 weeks after the intervention and at 12 weeks post-intervention.

**Table 1:** Characteristics of included randomized controlled trials.

Study	VR type	Experimental group							Control group							Assessment time points
		Intervention	Duration (min/session)	Frequency (times/week)	Total VR (wks)	Sample size	Age (year)	Male (%)	Intervention	Duration (min/session)	Frequency (times/week)	Total (wks)	Sample size	Age (year)	Male (%)	
Ain et al. (34)	Non-immersive gaming console: Kinect	Xbox Kinect training	35–40	5	6	25	57.48 ± 10.60	92.00	Conventional exercise training	35–40	5	6	25	57.68 ± 10.43	76.00	Before and after 6 weeks of intervention
Choi et al. (35)	Non-immersive gaming console: Nintendo Wii	Wii VR	30	5	4	10	64.30 ± 10.30	50.00	OT	30	5	4	10	64.70 ± 11.30	50.00	Before and after 4 weeks of intervention
da Silva Ribeiro et al. (36)	Non-immersive gaming console: Nintendo Wii	Wii VR	60	2	8	15	53.70 ± 6.10	33.30	CPT	60	2	8	15	52.80 ± 8.60	40.00	Before and after 8 weeks of intervention
Kim et al. (37)	Non-immersive gaming console: Kinect	Kinect VR + OT	30 + 30	5	2	12	56.70 ± 17.80	58.30	Sham VR + OT	30+ 30	5	2	11	57.20 ± 15.0	90.90	Before and after 2 weeks of intervention, 4-week follow-up
Kottink et al. (38)	NIVR	NIVR	30	3	6	8	65.30 ± 6.50	50.00	Conventional reach exercises	30	3	6	10	58.40 ± 14.80	90.00	Before and after 6 weeks of intervention, 4-week follow-up
Mekbib et al. (39)	IVR: head-mounted device	Customized IVR	60	4	2	12	52.17 ± 13.26	75.00	COT	60	4	2	11	61.00 ± 7.69	72.73	Before and after 2 weeks of intervention
Kong et al. (40)	Non-immersive gaming console: Nintendo Wii	Wii VR	60	4	3	35	58.10 ± 9.10	81.80	CT	60	4	3	35	59.00 ± 13.60	71.40	Before and after 3 weeks of intervention, 4 weeks after intervention, 12 weeks after intervention
Hsu et al. (41)	IVR: head-mounted device	Immersive mirror feedback therapy	30	2	9	18	52.9 ± 11.8	44.44	COT	30	2	9	17	56.90 ± 13.00	29.41	Before and after 9 weeks of intervention, 3-week follow-up
Henrique et al. (42)	IVR: head-mounted device	Motion Rehab AVE 3D VR	30	2	12	16	76.19 ± 10.09	43.75	CPT	30	2	12	15	76.20 ± 10.41	46.67	Before and after 12 weeks of intervention
Huang et al. (43)	IVR: head-mounted device	HTC VIVE VR	60 min × 2	3	16	15	50.80 ± 12.32	40.00	COT	60 min × 2	3	16	15	58.33 ± 11.22	27.00	Before and after 16 weeks of intervention
Ogun et al. (44)	IVR: head-mounted device	3D IVR with leap motion	60	3	6	33	61.48 ± 10.92	84.80	CT + sham virtual reality	45+ 15	3	6	32	59.75 ± 8.07	71.90	Before and after 6 weeks of intervention
Hung et al. (45)	Non-immersive gaming console: Kinect	Kinect2Screatch training	30	2	12	17	55.32 ± 15.29	70.59	Therapist-based training	30	2	12	16	58.54 ± 14.36	75.00	Before and after 12 weeks of intervention, 12-week follow-up
Lee et al. (46)	Non-immersive gaming console: Kinect	Kinect VR	30	3	8	13	66.46 ± 7.26	76.92	Group-based rehabilitation	30	3	8	13	69.92 ± 7.18	61.54	Before and after 8 weeks of intervention
Junior et al. (47)	Non-immersive gaming console: Nintendo Wii	Wii VR	50	2	8	11	55.50 ± 9.60	54.60	Conventional PNF	50	2	8	15	58.20 ± 7.70	53.40	Before and after 8 weeks of intervention
Teremetz et al. (48)	Non-immersive gaming console: Nintendo Wii	Wii VR	60	3	4	19	55.80 ± 12.66	69.00	CT	60	3	4	21	56.20 ± 12.52	53.00	Before and after 4 weeks of intervention
Kiper et al. (49)	NIVR	VR rehabilitation system	120	5	4	68	62.50 ± 15.20	54.40	CT	120	5	4	68	66.00 ± 12.90	63.20	Before and after 4 weeks of intervention
Oh et al. (50)	NIVR	Joystim VR	30	3	6	17	57.40 ± 12.20	38.70	CT	30	3	6	14	52.60 ± 10.70	29.00	Before and after 6 weeks of intervention, 4-week follow-up
Piron et al. (51)	NIVR	Reinforced feedback in virtual environment	60	5	4	27	58.80 ± 8.30	62.96	CT	60	5	4	23	62.20 ± 9.75	52.17	Before and after 4 weeks of intervention
Shin et al. (52)	NIVR	RAPAEL system+ CT	30 + 30	5	4	24	57.20 ± 10.30	79.20	CT	60	5	4	22	59.80 ± 13.00	77.30	Before and after 4 weeks of intervention, 4-week follow-up
Shin et al. (53)	NIVR	RAPAEL system+ CT	30 + 30	5	4	20	57.00 ± 12.78	50.00	CT	60	5	4	16	63.69 ± 8.58	43.75	Before and after 4 weeks of intervention, 4-week follow-up
Anwar et al. (54)	Non-immersive gaming console: Nintendo Wii	Nintendo Wii Games	60	3	6	34	51.50 ± 7.20	/	PT	60	3	6	34	51.35 ± 5.78	/	Before and after 6 weeks of intervention
Huang et al. (55)	IVR: head-mounted device	IVR + CR	30 + 30	5	3	20	63.30 ± 14.30	65.00	CR	60	5	3	20	65.10 ± 6.10	55.00	Before and after 3 weeks of intervention, 12-week follow-up
Shin et al. (56)	NIVR	VR + OT	30 + 30	5	4	16	53.30 ± 11.80	68.75	OT	60	5	4	16	54.60 ± 13.40	81.25	Before and after 4 weeks of intervention
Alves et al. (57)	Non-immersive gaming console: Nintendo Wii	Nintendo Wii Games	75	5	2	17	55.05 ± 11.52	64.70	CT	75	5	2	10	60.16 ± 13.19	70.00	Before and after 2 weeks of intervention
Assadi et al. (58)	Non-immersive gaming console: Nintendo Wii	Nintendo Wii Games	60	3	4	10	63.95 ± 7.31	50.00	CT	60	3	4	10	71.92 ± 14.19	50.00	Before and after 4 weeks of intervention
In et al. (59)	NIVR	VR + CT	30	5	4	11	63.45 ± 11.78	63.64	CT	30	5	4	8	64.50 ± 12.69	50.00	Before and after 4 weeks of intervention
Johnson et al. (60)	NIVR	Virtual therapy	45	2	8	28	64.70 ± 13.90	60.71	UC	45	2	8	30	59.30 ± 15.60	46.67	Before and after 8 weeks of intervention
Kiper et al. (61)	NIVR	VR rehabilitation system + TR	60 + 60	5	4	23	63.10 ± 9.50	61.00	TR	120	5	4	21	65.50 ± 14.20	71.00	Before and after 4 weeks of intervention
Lam et al. (62)	NIVR	Bilateral movement-based computer games + CR	210	2	8	47	65.1 ± 10.02	57.40	Video-directed exercise + CR	210	2	8	46	66.00 ± 9.00	60.90	Before and after 8 weeks of intervention, 4-week follow-up
Levin et al. (63)	NIVR	NIVR	45	3	3	8	58.10 ± 14.60	50.00	CT	45	3	3	6	59.80 ± 15.10	50.00	Before and after 3 weeks of intervention, 4-week follow-up
Park et al. (64)	NIVR	VR rehabilitation system	30	5	4	13	53.50 ± 13.00	53.80	Control	30	5	4	13	51.50 ± 16.70	61.50	Before and after 4 weeks of intervention, 4-week follow-up
Piron et al. (65)	NIVR	VR rehabilitation system	60	5	5–7	25	61.50 ± 9.40	68.00	CR	60	5	5–7	13	61.20 ± 6.60	61.54	Before and after 5–7 weeks of intervention
Turolla et al. (66)	NIVR	VR rehabilitation system+ ULC	120	5	4	263	60.20 ± 14.30	60.00	ULC	120	5	4	113	65.40 ± 12.50	64.00	Before and after 4 weeks of intervention
Turolla et al. (67)	NIVR	VR rehabilitation system	60	5	4	15	59.10 ± 8.60	92.30	CT	60	5	4	15	61.30 ± 10.50	53.33	Before and after 4 weeks of intervention

VR, Virtual reality; NIVR, Non-immersive virtual reality; IVR, Immersive virtual reality; CR, Conventional Rehabilitation; TR, Traditional Rehabilitation; OT, Occupational therapy; CPT, Conventional physiotherapy; COT, Conventional occupational therapy; CT, Conventional therapy; PT, Physical therapy; UC, Usual care alone; ULC, Upper limb conventional; PNF, Proprioceptive neuromuscular facilitation.

### 3.3 Risk of bias

**Figure 2:**
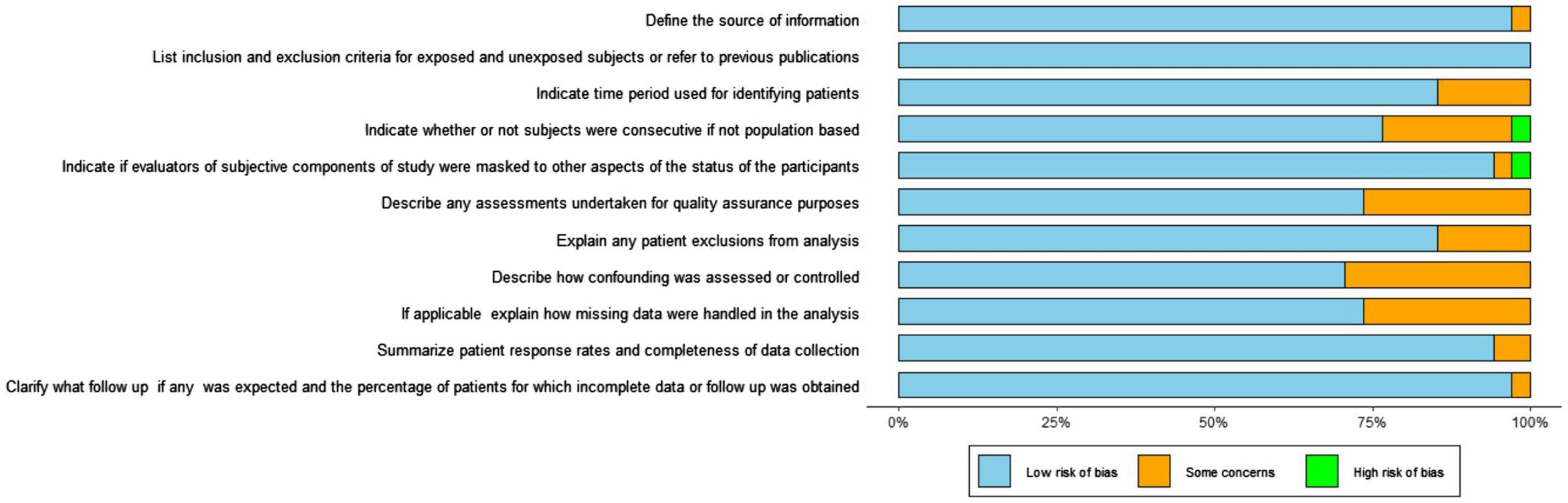
The quality assessment of the risk of bias summary of included studies using RoB-2.

The risk-of-bias assessment in the RCTs (Figure 2) demonstrated a low risk of bias across the analyzed trials.

### 3.4 Results of network meta-analysis

#### 3.4.1 Network diagram

**Figure 3:**
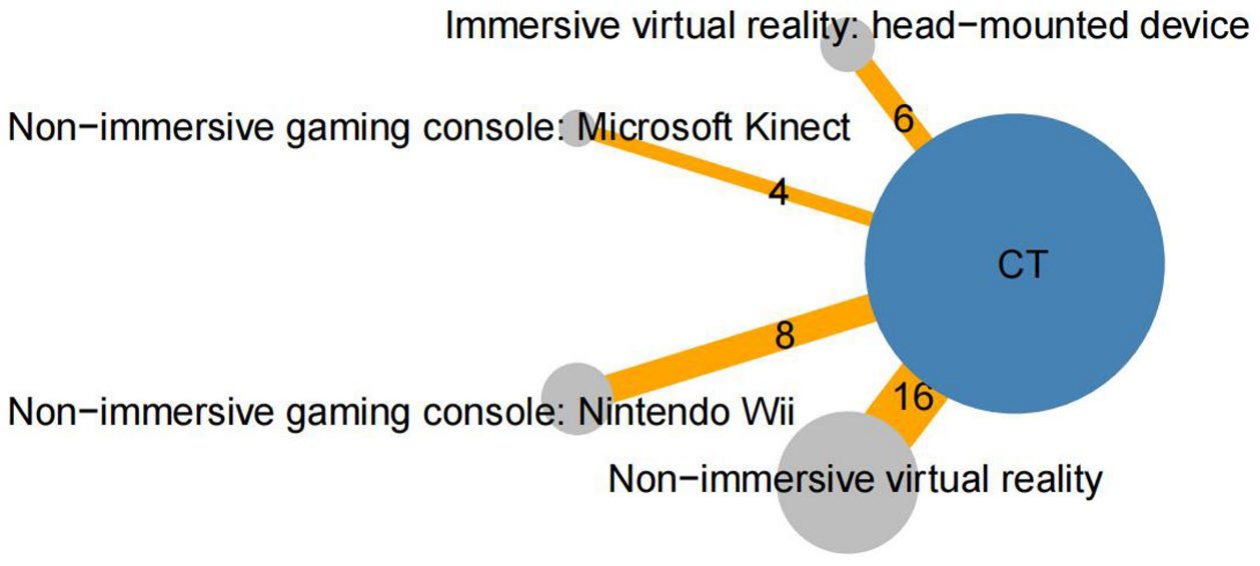
Network of randomized controlled trials (RCTs) comparing different virtual reality modalities for upper limb recovery after stroke. The size of each circle represents the number of participants in the trial, while the thickness of the connecting lines corresponds to the number of participants directly comparing the two treatments. The numbers indicate the number of trials contributing to each treatment comparison. CT, Conventional therapy.

The network diagram is displayed in Figure 3. Six studies (39, 41–44, 55) compared head-mounted devices to CT, while 16 studies compared NIVR systems to CT (38, 49–53, 56, 59–67). A total of 12 studies utilized non-immersive gaming consoles; four (34, 37, 45, 46) compared Microsoft Kinect to CT, and eight (35, 36, 40, 47, 48, 54, 57, 58) compared Nintendo Wii to CT. Among these interventions, CT had the largest sample size (759 participants), followed by NIVR (613 participants), Nintendo Wii (151 participants), head-mounted devices (114 participants), and Microsoft Kinect (67 participants).

#### 3.4.2 Consistency and inconsistency

In terms of model fit, both fixed-effects and random-effects models were examined. As illustrated in Supplementary Figure S1, the random-effects model demonstrated a superior fit, with lower DIC values and visually minimized leverage outliers, showed a better fit for the data than the fixed-effects model. Consequently, the random-effects model was selected for the final analysis. A Gelman–Rubin–Brooks plot was utilized to evaluate the convergence, which revealed that the simulations were valid, as the PSRF was <1.05 (Supplementary Figure S2, Gelman convergence plot).

Secondly, as consistency is a key assumption in NMA, we evaluated the model’s inconsistency by fitting a random-effects inconsistency model and comparing it with our random-effects consistency model. Supplementary Figure S3 illustrates the consistency and inconsistency models for FMUE. The consistency model demonstrated a marginally lower DIC value, suggesting a superior fit.

#### 3.4.3 Treatment ranking analysis

**Figure 4:**
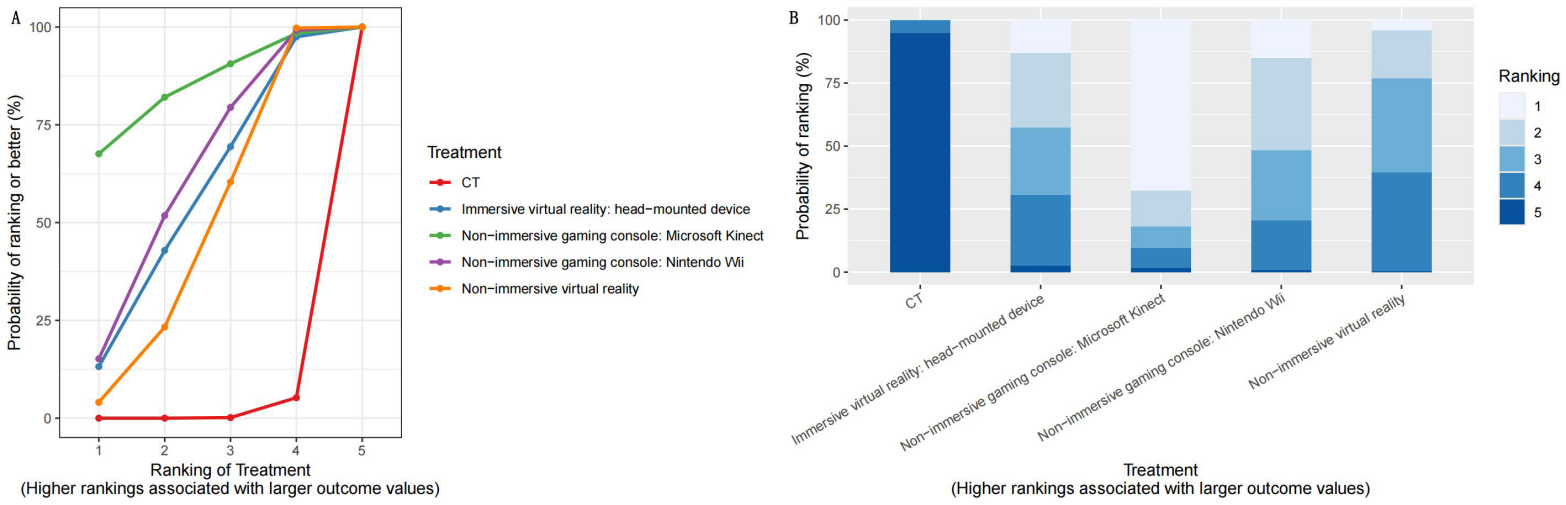
Results of the network meta-analysis of different modalities for upper limb recovery after stroke. **(A)** SUCRA plot for FMUE. **(B)** Rankogram plot of interventions for FMUE. SUCRA, surface under the cumulative ranking, represents the probability of ranking for each treatment, as shown in the graphs; FMUE, The Fugl-Meyer Upper Extremity; CT, Conventional therapy.

A treatment rank probability analysis was conducted to compare the posterior probabilities of each treatment, determining their relative rankings for FMUE outcomes. Additionally, we generated SUCRA plots to visually illustrate the percentage probability of rankings. For FMUE, examining the SUCRA plot (Figure 4A) and treatment rank plot (Figure 4B), reveal that non-immersive gaming consoles of Microsoft Kinect (SUCRA value 0.847) was associated with the highest probability of improving UL function in stroke patients, thereby ranking as the most effective treatment. This was followed by non-immersive gaming console of Nintendo Wii (SUCRA value 0.614), Immersive VR: head-mounted device (SUCRA value 0.557), and Non-immersive VR (SUCRA value 0.469). CT was identified as the least effective treatment, indicating minimal impact on improving FMUE.

#### 3.4.4 Relative effectiveness of treatment

**Figure 5:**
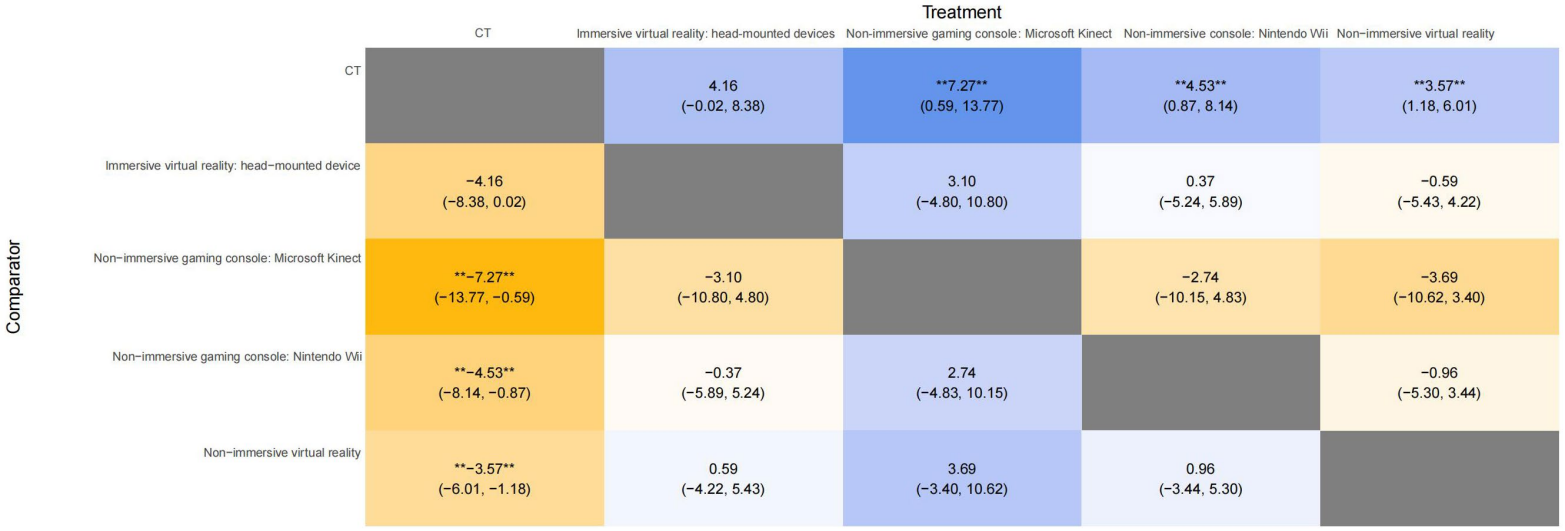
League table for all treatment in the network for FMUE. The symbol ** in the figure indicates significant differences between the treatments (*p* < 0.05). FMUE, The Fugl-Meyer Upper Extremity; CT, Conventional therapy.

A league plot was generated to provide a comprehensive summary of the NMA results, comparing the effectiveness of various interventions in improving the FMUE score. The symbols (**) indicate statistically significant differences between treatments and comparators at a 95% confidence level, with *p* < 0.05. As shown in Figure 5, non-immersive gaming consoles, including the Microsoft Kinect and Nintendo Wii, significantly improved UL function compared to CT. Specifically, the non-immersive gaming consoles of Microsoft Kinect yielded MD of 7.27 (95% CI: 0.59 to 13.77), indicating a significant benefit (*p* < 0.05) over CT. While the non-immersive gaming console Nintendo Wii showed a significant improvement (*p* < 0.05) with MD of 4.53 (95% CI: 0.87 to 8.14). In addition, NIVR was also found to have a moderate benefit, with an MD of 3.57 (95% CI: 1.18 to 6.01) and *p* < 0.05. In contrast, IVR head-mounted devices showed no statistically significant differences in outcomes, with MD of 4.16 (95% CI: −0.02 to 8.38).

**Figure 6:**
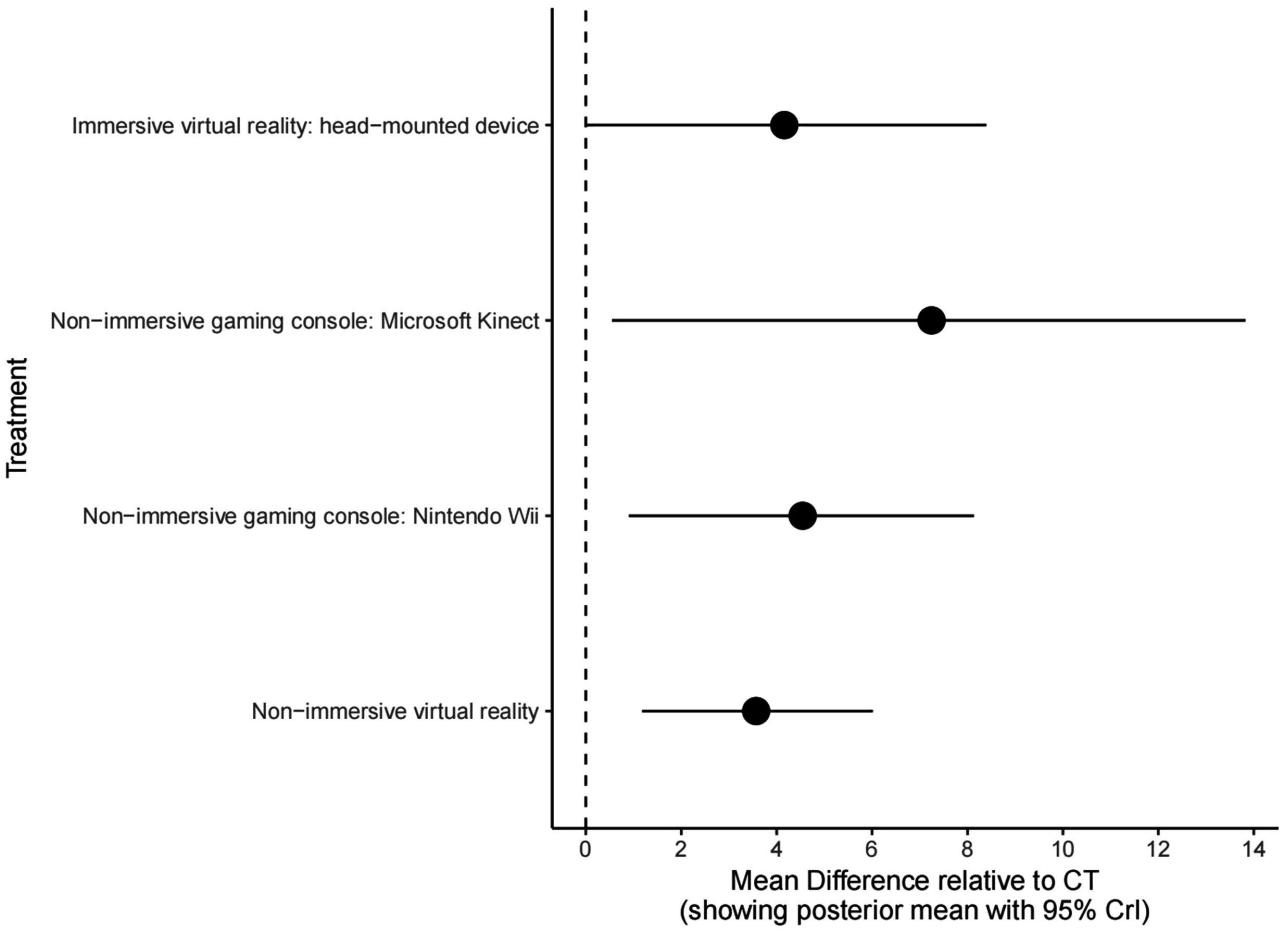
Forest plot for all treatments compared to conventional therapy as reference for FMUE. FMUE, The Fugl-Meyer Upper Extremity; CT, Conventional therapy.

To obtain the visualization of the MD differences with a 95% CI between interventions, forest plots were performed for FMUE (Figure 6). Both plots show approximately the same results, indicating that non-immersive gaming consoles of Microsoft Kinect interventions significantly improved FMUE, followed by the non-immersive gaming console Nintendo Wii intervention, and then NIVR.

## 4 Discussion

### 4.1 Principal findings

This systematic review and NMA examined 34 RCTs encompassing 1,704 stroke survivors to assess VR interventions with varying immersion levels on UL motor function using the FMUE scale. The analysis indicates that non-immersive gaming consoles of Microsoft Kinect, demonstrated the most substantial improvement in UL function, followed by the Nintendo Wii and other NIVR systems. Conversely, IVR systems did not demonstrate significant superiority over CT.

### 4.2 Validity of evidence

The findings are supported by a rigorous methodological approach. All included studies ensured comparable intervention frequency and duration between VR and CT groups, minimizing potential confounding factors. This analysis included only high-quality RCTs, with risk-of-bias assessments confirming low risk across all domains. The FMUE scale, consistently used as the primary outcome measure, provided a reliable, stroke-specific evaluation of UL recovery (68). Additionally, the Bayesian NMA facilitated the simultaneous comparison and ranking of multiple interventions, enhancing the robustness of the conclusions.

### 4.3 Superiority of non-immersive gaming consoles

Our findings demonstrate the significant benefits of non-immersive gaming consoles of Microsoft Kinect in UL rehabilitation. Microsoft Kinect’s precise motion-sensing technology enhances interactivity, fostering greater patient engagement and motivation—crucial factors in motor relearning (69, 70). Moreover, its personalized training content and accessibility render it suitable for both clinical and home-based applications (71).

While the Nintendo Wii also provides interactivity, its limited motion capture accuracy and less advanced game design reduce its effectiveness in stroke rehabilitation (72, 73). Additionally, Microsoft Kinect has been shown to improve activities of daily living and cognitive engagement, further corroborating its therapeutic potential (74). Conversely, IVR systems, despite their immersive experiences, encounter substantial obstacles to widespread implementation due to hardware complexity, high costs, and patient discomfort, including motion sickness (75). These factors may impede training duration and adherence among elderly or physically frail patients (76).

Variations in UL rehabilitation outcomes may be attributed to differences in motion capture technology and task design. Microsoft Kinect (34, 37, 45, 46) systems employ camera-based motion tracking, enabling natural UL movements, while the Wii relies on handheld controllers, which potentially restrict distal upper extremity engagement (77).

### 4.4 Comparison with previous literature

Our findings are consistent with those of Soleimani et al. (78), who similarly demonstrated the significantly improve in UL motor recovery post-stroke through VR interventions. However, Soleimani’s meta-analysis was constrained by pairwise comparisons, limiting the evaluation of different VR modalities. In contrast, our study employed the NMA framework, enabling for direct comparisons across multiple VR modalities and offering a more comprehensive ranking of interventions.

To the best of our knowledge, only one other NMA has assessed the efficacy of various VR interventions for UL motor function, involving 20 RCTs with 813 participants (22). However, our analysis identified non-immersive gaming consoles of Microsoft Kinect, as the most effective, while the previous NMA favored immersive VR. These differences may be attributed to advancements in NIVR technology and our analysis of a larger, more recent dataset.

### 4.5 Limitations

Several limitations should be considered when interpreting our findings. Firstly, the inability to differentiate between acute, subacute, and chronic stroke phases constrains the applicability of our results to specific rehabilitation stages. Secondly, the lack of long-term data limits our understanding of the sustainability of VR interventions beyond the post-intervention period. Thirdly, the absence of direct head-to-head comparisons among different VR systems restricts the robustness and comprehensiveness of the conclusions, as indirect comparisons may introduce additional uncertainties. However, we included studies with highly similar designs and population characteristics to ensure the validity and consistency of the indirect comparisons. Additionally, we employed a consistency model to minimize potential biases arising from the absence of direct comparisons. In light of these limitations, the findings of our network analysis should be interpreted with caution. We recommend that subsequent studies validate our findings through direct comparisons.

### 4.6 Implications for clinical practice and future research

This study underscores the cost-effectiveness and accessibility of non-immersive gaming consoles of Microsoft Kinect interventions for stroke rehabilitation. These platforms offer an interactive environment that promotes patient engagement and adherence—key factors for sustained recovery. Future research should focus on assessing long-term outcomes and conducting comprehensive cost-effectiveness analyses. In future research, more refined classification methods should be employed, or studies should focus on individual stages of stroke recovery to enhance the applicability of the results to specific rehabilitation phases. Additionally, future studies should prioritize the evaluation of long-term outcomes and conduct comprehensive cost-effectiveness analyses to gain a better understanding of the sustainability of VR interventions during the post-intervention period.

## 5 Conclusion

This NMA highlights the significant benefits of non-immersive gaming consoles of Microsoft Kinect, in enhancing UL motor function among stroke survivors. The findings support the integration of non-immersive gaming consoles of Microsoft Kinect interventions into clinical practice. However, our analysis also reveals that CT provides the least improvement in upper limb motor function, underscoring the need for more engaging and interactive rehabilitation tools. This comparison between the most effective (Microsoft Kinect) and least effective interventions clearly demonstrates the potential of gaming consoles in stroke rehabilitation.

## Data Availability

The original contributions presented in the study are included in the article/Supplementary material, further inquiries can be directed to the corresponding authors.

## Supplementary material

SUPPLEMENTARY FIGURE S1 Leverage plots and DIC for fixed and random effects models for FMUE.

SUPPLEMENTARY FIGURE S2 Gelman convergence plot.

SUPPLEMENTARY FIGURE S3 Leverage plots and DIC for consistency and inconsistency model FMUE.
