# Supplementary figures and images for: Effects of virtual reality with different modalities on upper limb recovery: a systematic review and network meta-analysis on optimizing stroke rehabilitation
Source: Front Neurol. 2025 Apr 1;16:1544135. doi: 10.3389/fneur.2025.1544135 (PMC11996652; doi:10.3389/fneur.2025.1544135)

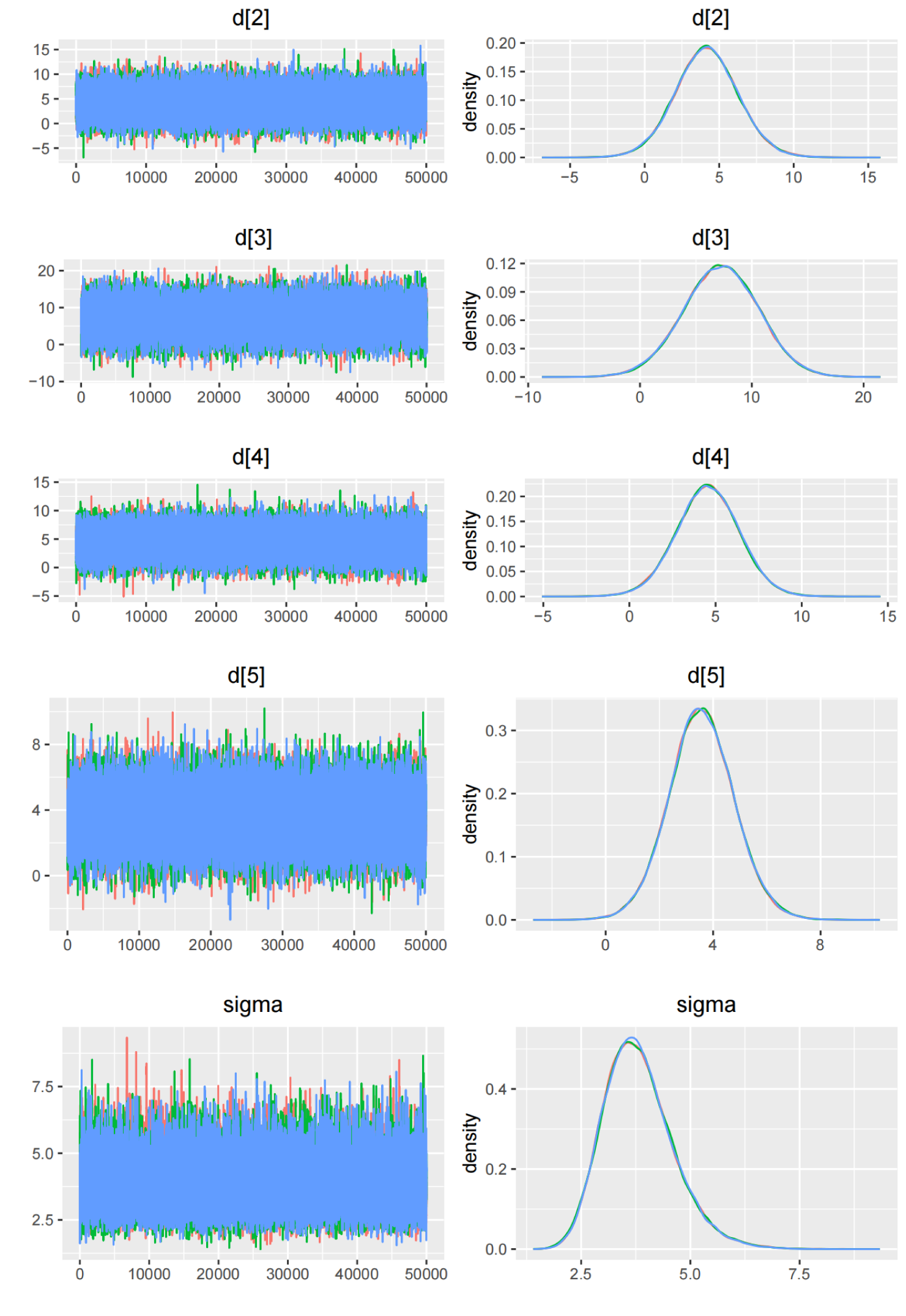

Supplement: SUPPLEMENTARY FIGURE S1 — Leverage plots and DIC for fixed and random effects models for FMUE. [file Image_1.tif]

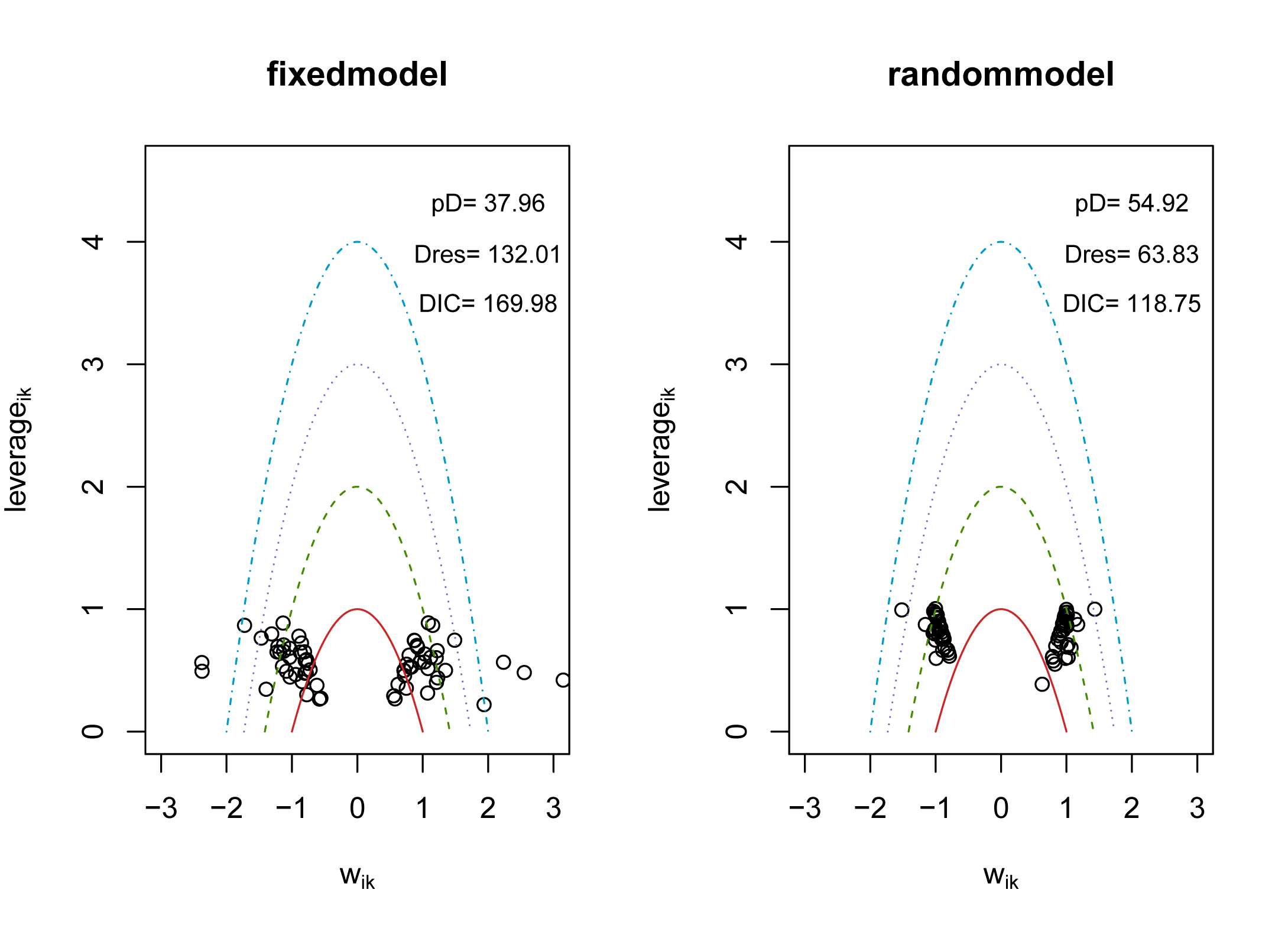

Supplement: SUPPLEMENTARY FIGURE S2 — Gelman convergence plot. [file Image_2.tif]

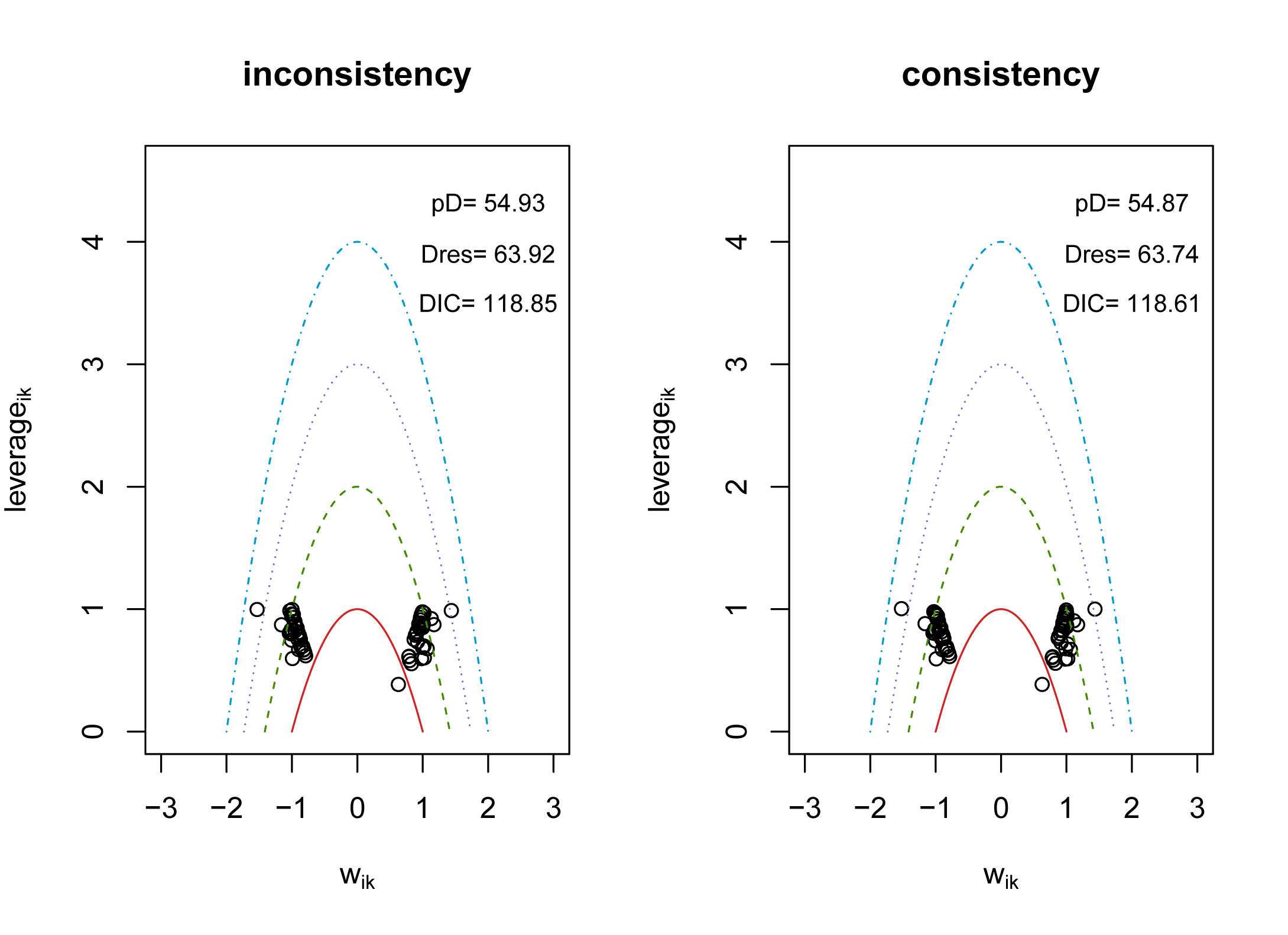

Supplement: SUPPLEMENTARY FIGURE S3 — Leverage plots and DIC for consistency and inconsistency model FMUE. [file Image_3.tif]
