# Supplementary material for: Effects of virtual reality with different modalities on upper limb recovery: a systematic review and network meta-analysis on optimizing stroke rehabilitation
Source: Front Neurol. 2025 Apr 1;16:1544135. doi: 10.3389/fneur.2025.1544135 (PMC11996652; doi:10.3389/fneur.2025.1544135)
Supplement: Supplementary file 4 [file Table_1.doc]

**S1_Table. Search strategy.**

**PubMed search strategy**

| Number | Terms |
| --- | --- |
| #1 | "cerebrovascular disorders" [MeSH Terms] |
| #2 | stroke*[Title/Abstract] OR poststroke*[Title/Abstract] OR cva*[Title/Abstract])) |
| #3 | cerebrovascular*[Title/Abstract] OR cerebral vascular[Title/Abstract] |
| #4 | cerebral[Title/Abstract] OR cerebellar[Title/Abstract] OR brain* [Title/Abstract] OR vertebrobasilar[Title/Abstract] |
| #5 | infarct*[Title/Abstract] OR ischaemi* [Title/Abstract] OR ischemi* [Title/Abstract] OR thrombo* [Title/Abstract] OR emboli*[Title/Abstract] OR apoplexy[Title/Abstract]))) |
| #6 | cerebral[Title/Abstract] OR intracerebral[Title/Abstract] OR intracranial[Title/Abstract] |
| #7 | haemorrhage[Title/Abstract] OR hemorrhage [Title/Abstract] OR bleed*[Title/Abstract]) |
| #8 | #4 AND #5 |
| #9 | #6 AND #7 |
| #10 | #1 OR #2 OR #3 OR #8 OR #9 |
| #11 | Upper Extremity [MeSH Terms] |
| #12 | (upper limb*[Title/Abstract] OR upper extremit*[Title/Abstract] OR arm[Title/Abstract] OR arms[Title/Abstract] OR shoulder[Title/Abstract] OR shoulders[Title/Abstract] OR hand[Title/Abstract] OR hands[Title/Abstract] OR axilla*[Title/Abstract] OR elbow*[Title/Abstract] OR forearm*[Title/Abstract] OR finger*[Title/Abstract] OR wrist*[Title/Abstract]) |
| #13 | #11 OR #12 |
| #14 | user-computer interface[MeSH Terms] |
| #15 | computers[MeSH Terms] OR microcomputers[MeSH Terms] OR computer systems[MeSH Terms] OR software[MeSH Terms] |
| #16 | (computer simulation[MeSH Terms] OR computer-assisted instruction[MeSH Terms] OR therapy, computer-assisted[MeSH Terms]) |
| #17 | (computer graphics[MeSH Terms] OR video games[MeSH Terms]) |
| #18 | (virtual reality[MeSH Terms] OR virtual reality exposure therapy[MeSH Terms]) |
| #19 | (virtual reality* [Title/Abstract] OR virtual-reality* [Title/Abstract] OR VR [Title/Abstract]) |
| #20 | (virtual [Title/Abstract] AND (environment*[Title/Abstract] OR object*[Title/Abstract] OR treatment*[Title/Abstract] OR system*[Title/Abstract] OR program*[Title/Abstract] OR rehabilitation*[Title/Abstract] OR therap*[Title/Abstract] OR driving[Title/Abstract] OR drive* [Title/Abstract] OR car[Title/Abstract] OR tunnel[Title/Abstract] OR vehicle[Title/Abstract])) |
| #21 | (computer[Title/Abstract] AND (simulat*[Title/Abstract] OR graphic*[Title/Abstract] OR game*[Title/Abstract] OR interact*[Title/Abstract])) |
| #22 | (computer[Title/Abstract] AND assist*[Title/Abstract] AND (therap*[Title/Abstract] OR treat*[Title/Abstract])) |
| #23 | (computer [Title/Abstract] AND generat*[Title/Abstract] AND (environment*[Title/Abstract] OR object*[Title/Abstract])) |
| #24 | (video games[MeSH Terms]) |
| #25 | (videogames[Title/Abstract] OR "video games"[Title/Abstract] OR "video-games"[Title/Abstract] OR "serious videogames"[Title/Abstract] OR "serious gam*"[Title/Abstract] OR "serious video games"[Title/Abstract] OR "exergames"[Title/Abstract] OR "exergaming"[Title/Abstract] OR "active videogames"[Title/Abstract]) |
| #26 | ("video game*"[Title/Abstract] OR "video gaming"[Title/Abstract] OR "gaming console*"[Title/Abstract] OR "interactive game"[Title/Abstract] OR "interactive gaming"[Title/Abstract] OR "Nintendo Wii"[Title/Abstract] OR Kinect[Title/Abstract] OR "gaming program*"[Title/Abstract]) |
| #27 | (haptics[Title/Abstract] OR "haptic device*"[Title/Abstract]) |
| #28 | (simulat*[Title/Abstract] AND (environment*[Title/Abstract] OR object*[Title/Abstract] OR event*[Title/Abstract] OR driving[Title/Abstract] OR drive*[Title/Abstract] OR car[Title/Abstract] OR tunnel[Title/Abstract] OR vehicle[Title/Abstract])) |
| #29 | (user[Title/Abstract] AND computer[Title/Abstract] AND interface[Title/Abstract]) |
| #30 | #14 OR #15 OR #16 OR #17 OR #18 OR #19 OR #20 OR #21 OR #22 OR #23 OR #24 OR #25 OR #26 OR #27 OR #28 #29 |
| #31 | #10 AND #13 AND #30 |
| #32 | (randomized controlled trial[pt] OR controlled clinical trial[pt] OR randomized[tiab] OR placebo[tiab] OR clinical trials as topic[mesh:noexp] OR randomly[tiab] OR trial[ti]) NOT (animals [mh] NOT (humans [mh] AND animals[mh])) |
| #33 | #31 AND #32 |
